# Integration of quantitated expression estimates from polyA-selected and rRNA-depleted RNA-seq libraries

**DOI:** 10.1186/s12859-017-1714-9

**Published:** 2017-06-13

**Authors:** Stephen J. Bush, Mary E. B. McCulloch, Kim M. Summers, David A. Hume, Emily L. Clark

**Affiliations:** The Roslin Institute and Royal (Dick) School of Veterinary Studies, University of Edinburgh, Easter Bush, Midlothian, EH25 9RG UK

**Keywords:** RNA-seq, Gene expression, Expression atlas, polyA-selection, rRNA-depletion, Kallisto

## Abstract

**Background:**

The availability of fast alignment-free algorithms has greatly reduced the computational burden of RNA-seq processing, especially for relatively poorly assembled genomes. Using these approaches, previous RNA-seq datasets could potentially be processed and integrated with newly sequenced libraries. Confounding factors in such integration include sequencing depth and methods of RNA extraction and selection. Different selection methods (typically, either polyA-selection or rRNA-depletion) omit different RNAs, resulting in different fractions of the transcriptome being sequenced. In particular, rRNA-depleted libraries sample a broader fraction of the transcriptome than polyA-selected libraries. This study aimed to develop a systematic means of accounting for library type that allows data from these two methods to be compared.

**Results:**

The method was developed by comparing two RNA-seq datasets from ovine macrophages, identical except for RNA selection method. Gene-level expression estimates were obtained using a two-part process centred on the high-speed transcript quantification tool Kallisto. Firstly, a set of reference transcripts was defined that constitute a standardised RNA space, with expression from both datasets quantified against it. Secondly, a simple ratio-based correction was applied to the rRNA-depleted estimates. The outcome is an almost perfect correlation between gene expression estimates, independent of library type and across the full range of levels of expression.

**Conclusion:**

A combination of reference transcriptome filtering and a ratio-based correction can create equivalent expression profiles from both polyA-selected and rRNA-depleted libraries. This approach will allow meta-analysis and integration of existing RNA-seq data into transcriptional atlas projects.

**Electronic supplementary material:**

The online version of this article (doi:10.1186/s12859-017-1714-9) contains supplementary material, which is available to authorized users.

## Background

RNA sequencing (RNA-seq) – the unbiased sequencing of random cDNA fragments [1] – has many applications, the most common of which are to quantify expression level, identify allelic imbalance, and discover novel genes, transcripts and splice variants [2–4]. RNA-seq has benefitted from decreasing sequencing costs which, since 2008, have declined at a rate faster than expected given Moore’s law (which describes an exponential growth rate in computer hardware) [5]. As lower material costs help overcome limitations of breadth (diversity of samples sequenced) and depth (the ability to capture rare transcripts) in a given study, an increasing number of gene expression atlases – large-scale compendia of transcript abundance across a range of tissues and cell types – have been developed, including atlases for the rat [6], sheep [7], maize [8, 9], soybean [10], the string bean *Phaesolus vulgaris* [11], the pea *Pisum sativum* [12], and humans (as part of the ENCODE project) [13].

Despite the growing volume of RNA-seq data, it cannot easily be integrated into new projects because relative expression estimates depend upon methods of library preparation. Most notably, RNA-seq libraries vary in how RNA is extracted from the cell line or tissue (commonly using either phenol-chloroform or silica-gel column based methods [14]) and in how RNAs are selected for sequencing.

All RNA-seq libraries must effectively remove the highly abundant ribosomal RNA. The two most commonly used selection methods, polyA-selection (polyA+) and rRNA-depletion (ribo-minus), selectively omit a distinct set of RNAs (polyA- RNAs and rRNA, respectively) to sequence different fractions of the transcriptome [15]. These generate incompatible datasets, despite both methods performing the same task of removing highly abundant rRNAs to allow mRNA detection (rRNAs represent >80% of the total molecules in each sample [16]). polyA+ libraries exclude rRNA because rRNAs are not polyadenylated – they lack the polyA tail added post-transcriptionally to the 3′ end of almost all eukaryotic mRNAs (for its functional roles in mRNA stability, nucleocytoplasmic export and translation [17]). However, polyA+ selection also excludes, aside from rRNAs, many other mRNAs that are either polyA- (such as replication-dependent histones [18], various long non-coding RNAs (lncRNAs) [19, 20], and bacterial mRNA [21]) or bimorphic (existing in both the polyA- and polyA+ populations) [22, 23]. By contrast, ribo-minus libraries characterise both polyA+ and polyA- RNAs [24] – a more thorough profile of the transcriptome – but they also capture nascent (ongoing) transcription [25] and so contain a larger proportion of intronic sequence from pre-mature mRNA [26].

Current microarray platforms are sufficiently resilient that data generated from the same platform can be compared between labs, for example to generate a comprehensive expression atlas of human primary cells [27]. Similar consolidation of selected RNA-seq data would increase statistical and analytic power, and in the case of animal-based research, would meet the objective of reducing numbers used [28].

By contrast to microarrays, where each transcript is detected independently, in RNA-seq, expression is quantified in relative terms – for a given number of sequenced reads, the abundance of one transcript affects the relative abundance of the others. This can be accounted for by reporting expression level with a universal proportionality constant, i.e. in units of TPM, the number of transcripts per million. TPM is independent of transcript length (longer transcripts would otherwise generate more reads) and so is in principle comparable across samples [29]. However, its use assumes that a million reads from one experiment are equivalent to a million reads from another, which is clearly not the case with different library preparation methods.

RNA-seq data is commonly processed by aligning the sequenced reads to a reference genome, reconstructing transcripts from this set of alignments and quantifying their expression as a function of the reads aligned [30]. This alignment process is slow enough to cause an analytic bottleneck [31]. Alternative ‘lightweight’ algorithms (such as Sailfish [32], Salmon [33], RNA-skim [34], RapMap [35] and Kallisto [31]) utilise a pre-defined reference transcriptome, instead of a genome, for quantification. For instance, Kallisto builds an index of k-mers from a reference set of transcripts and then estimates expression level from the reads directly. Rather than aligning reads to each transcript (a time-consuming approximation, as alignments have gaps), k-mers (generated from transcripts) are instead matched exactly to each read [31].

To test whether polyA+ and ribo-minus datasets can be meaningfully combined, we used Kallisto to characterise the expression profiles of differentially selected RNA-seq datasets from libraries generated using each method – these have the same cell type (ovine bone marrow derived macrophages (BMDMs)), the same RNA and are run on the same sequencing platform (Illumina HiSeq 2500) at a depth of >25 million reads per sample. These samples form part of an ovine transcriptional atlas of multiple tissues and primary cells, developed at the Roslin Institute [36] as an expansion of efforts to provide functional annotation of the sheep genome [7]. The atlas combines the two methods, with a subset of samples based upon ribo-minus selection and sequenced at greater depth, to ensure comprehensive capture of alternative splice isoforms and non-polyadenylated transcripts.

We describe here the development and validation of an approach to data integration that enables quantitation of the sets of transcripts detected by both methods and their integration into a single dataset.

## Results

### RNA selection method affects estimates of expression level

In other species, including mice and humans [37], pigs [38] and horses [39], pure populations of macrophages can be generated by cultivating precursors in the macrophage growth factor, CSF1 (macrophage colony-stimulating factor), with these cells undergoing a profound and rapid change in gene expression when exposed to bacterial lipopolysaccharide (LPS). This response varies greatly between species. The same approach was developed for sheep, producing bone marrow derived macrophages (BMDMs) and analysing their response to LPS with the objective of a detailed comparative analysis of responses relative to other ruminants and species (manuscript in preparation). RNA was prepared from sheep BMDMs, before and after LPS treatment for 7 h, and libraries were prepared from the same RNA following either polyA+ selection or rRNA-depletion by standard protocols.

Expression was quantified using the high-speed transcript quantification tool Kallisto v0.43.0 [40] as the median TPM (transcripts per million) of 6 replicates (that is, BMDMs from 6 animals). Transcript-level abundances were summarised to the gene-level. This analysis requires that a k-mer index (k = 31) first be generated from a reference transcriptome: *Ovis aries* v3.1 (see Methods).

**Fig. 1:**
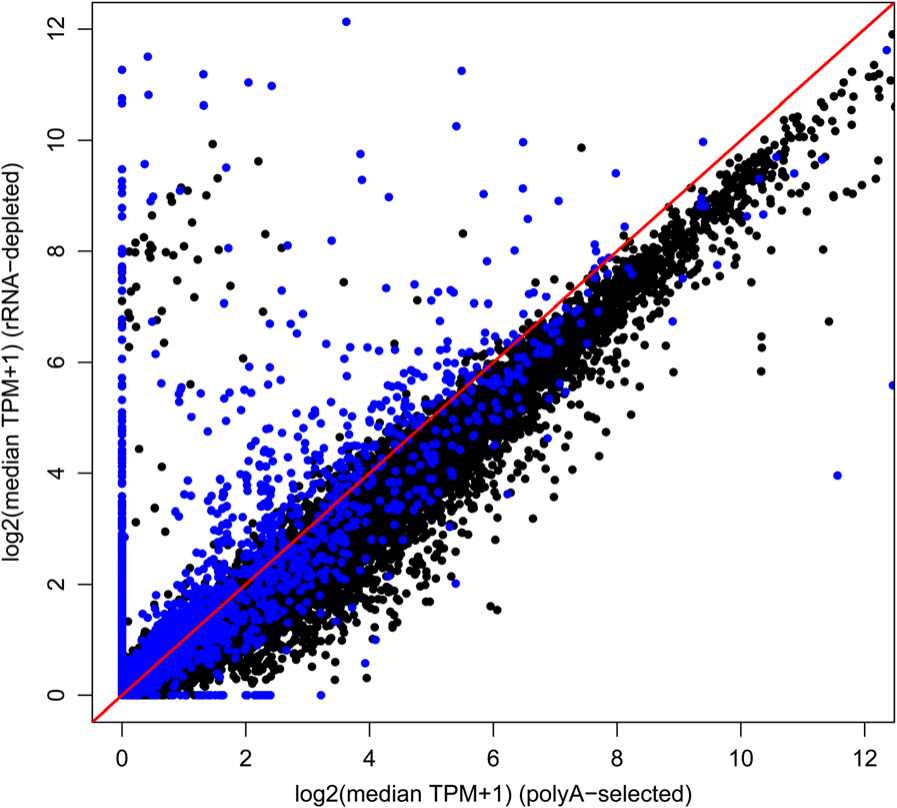
Differential transcriptome sampling by polyA+ and ribo-minus RNA selection methods leads to variance in TPM estimates. Each point is a gene, coloured by type: black points represent protein-coding genes, pseudogenes and processed pseudogenes (*n* = 21,211); blue points represent RNA genes (*n* = 5843). The line *y* = *x* is shown in red. As ribo-minus libraries capture RNA genes that polyA+ libraries do not (the line of points at *x* = 0), expression can be systematically underestimated for the remaining, mostly protein-coding, genes (Fig. 2). The data shown is for BMDMs prior to LPS stimulation. The same pattern was observed for BMDMs 7 h post-LPS stimulation (Additional file 1: Figure S1). The confounding effect of selection method can be corrected mathematically (Fig. 4)

**Fig. 2:**
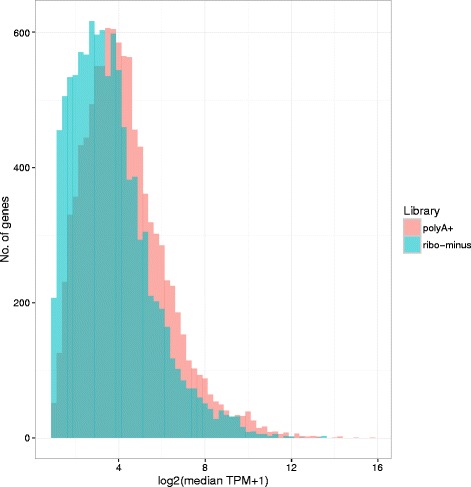
The expression level of protein-coding genes was systematically underestimated in ribo-minus compared to polyA+ libraries (mean for each distribution 3.65 and 4.31 TPM, respectively; T-test: *T* = 25.52, *p* < 2.2 × 10^−16^; Cliff’s delta = 0.22, indicative of a small effect). Only data from protein-coding genes with detectable expression (TPM > 1) in both library types is shown. Bins have width 0.25

Expression level estimates for all genes in the ovine BMDM transcriptome, both before and after treatment with LPS, are shown in Additional file 1: Table S1. Based upon data from unstimulated BMDMs, the expression levels estimated by alternative library preparation methods are correlated (Pearson’s *r* = 0.92, *p* < 2.2 × 10^−16^; see Fig. 1 and Additional file 1: Table S2), but ribo-minus libraries (which capture more RNA genes – that is, non-coding RNAs – than polyA+ libraries) systematically produce a lower estimate of the relative expression of protein-coding genes (T-test *p* < 2.2 × 10^−16^, Cliff’s delta = 0.22, interpreted as a small but significant effect; Fig. 2). Quantitatively similar findings were found when comparing data generated from LPS-stimulated BMDMs (Additional file 2: Figure S1).

Although both methods detect an equivalent number of genes in both conditions (TPM > 1 both pre- and post-LPS), the ribo-minus libraries capture 492 RNA genes that the polyA+ libraries do not (approx. 8% of the total number of known RNA genes, *n* = 5843) (Additional file 1: Table S3). Conversely, the polyA+ libraries capture 654 protein-coding genes that the ribo-minus libraries failed to detect above the TPM > 1 threshold (approx. 3% of the total). This differential gene detection is likely due to sampling noise. For the set of genes uniquely captured by only one selection method, expression is low both pre- and post-LPS stimulation. For polyA-selected libraries, 75% of these genes are <3 TPM; for rRNA-depleted libraries, < 8 TPM (Additional file 1: Tables S4 and S5). Notable exceptions were the highly expressed polyA- genes – such as the set of U1 spliceosomal RNAs [41], at >17,000 TPM – which could only be quantified here in rRNA-depleted libraries.

The summed TPM for genes specific to the ribo-minus libraries is in the region of 40-50,000 TPM, i.e. 4-5% of the total sequenced transcripts have no counterpart in a polyA+ library (Additional file 1: Table S5). The disparity between the two TPM distributions arises because k-mers unique to this subset of reads can be matched to transcripts in the Kallisto index (the reference transcriptome against which expression was quantified; see Methods).

K-mers do not always match transcripts on a one-to-one basis: numerous reads (and by extension, the k-mers derived from them) will map with equal validity to multiple loci. These reads are enriched amongst gene families which have many members with identical or near-identical sequence [42], and although they can be excluded from analysis to minimise ambiguity, this is at the cost of biologically meaningful data [43]. For both library types, approximately one fifth of the alignments are non-unique (Additional file 1: Table S6). To quantify expression level in these cases, Kallisto fractionally assigns the reads amongst the set of possible transcripts using an expectation-maximization algorithm (an iterative process whereby reads are assigned to transcripts and these assignments used to estimate abundance, with repetition until convergence [32]). As such, reference transcriptomes with a higher number of non-unique kmers will have a higher number of non-unique alignments, potentially skewing the TPM of certain genes – by fractionally assigning reads to their probable locations, some genes will be under- and some over-counted. Pseudogenes in particular are enriched for multi-mapped reads, as many also map to their functional counterpart [42].

**Fig. 3:**
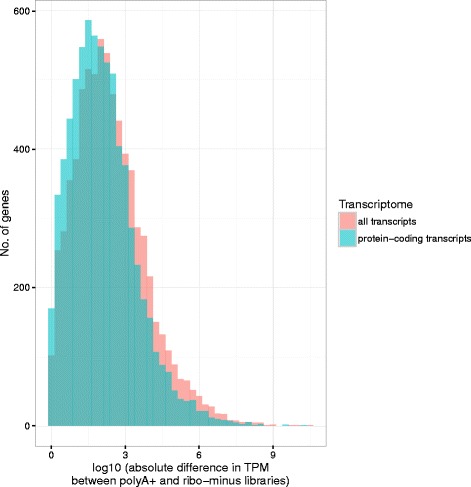
The absolute difference in TPM between polyA+ and ribo-minus libraries was reduced if expression was quantified against a smaller reference transcriptome. Although the difference was significant (T-test: *T* = 13.54, *p* < 2.2 × 10^−16^), it had a negligible effect on the shape of the distribution (Cliff’s delta = 0.13). The data used is from BMDMs prior to LPS stimulation. Only genes quantified at TPM > 1 against both reference transcriptomes are shown (*n* = 14,584). Bins have width 0.25

To eliminate the impact of multi-mapping and differential detection between library types, the reference transcriptome was filtered to remove those subsets of transcripts differentially detected by library type. This increased the fraction of unique k-mers distributed amongst the remaining transcripts. This correction was sufficient to reduce the effect size of the difference between the polyA+ and ribo-minus distributions to negligible levels (Additional file 1: Table S2). Furthermore, when expression was quantified using an index of protein-coding transcripts alone, > 600 extra genes met a threshold of 1 TPM compared to an index of all known transcripts (Additional file 1: Table S2). This is particularly important given many genes are only expressed at low levels – for instance, of the 17,180 genes with non-zero expression in unstimulated BMDMs (Additional file 1: Table S1), approximately half (8262 genes) have a TPM < 5. For these genes, there could still be a large effect in absolute terms from small differences in read/k-mer assignment (Fig. 3 and Additional file 1: Table S7).

### Expression levels estimated using ribo-minus libraries can be made equivalent to those using polyA+ libraries

**Fig. 4:**
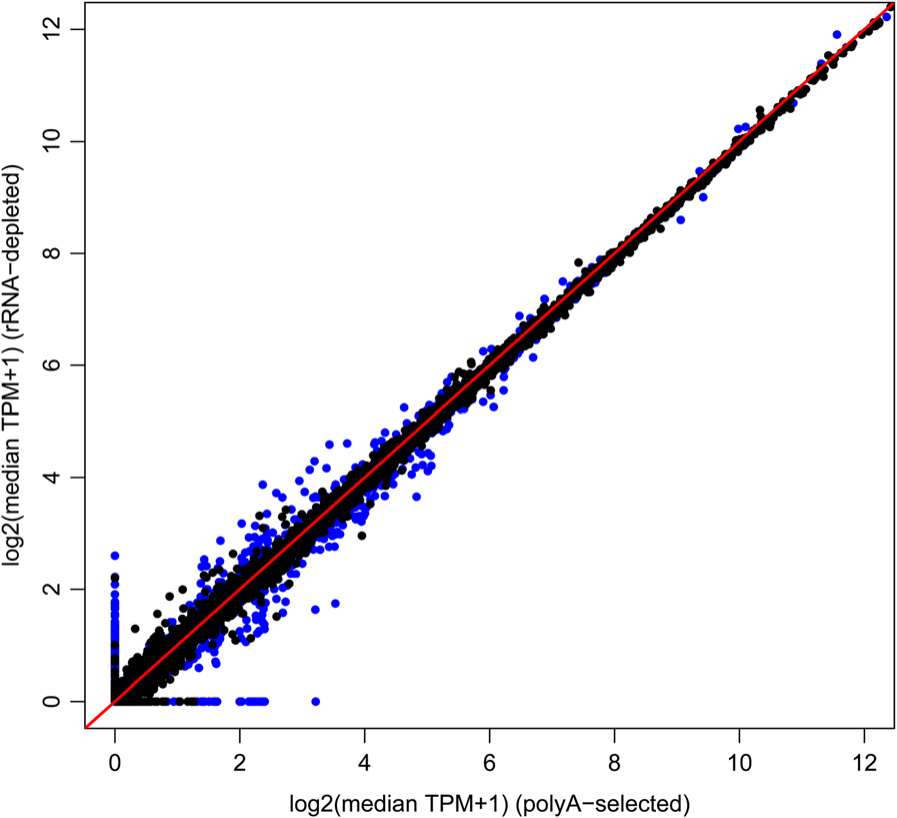
Variance in TPM estimates introduced by the differential transcriptome sampling of polyA+ and ribo-minus methods can be corrected mathematically. The same data is shown as in Fig. 1, except that all ribo-minus TPM estimates were multiplied by the ratio of the median TPM across all polyA+ libraries to the median TPM across all ribo-minus libraries. Should the median TPM across all ribo-minus libraries be 0, this ratio was considered 0 also. Each point is a gene, coloured by type: black points represent protein-coding genes, pseudogenes and processed pseudogenes; blue points represent RNA genes. The line *y* = *x* is shown in red. Pearson’s *r* = 0.998, *p* < 2.2 × 10^−16^

**Fig. 5:**
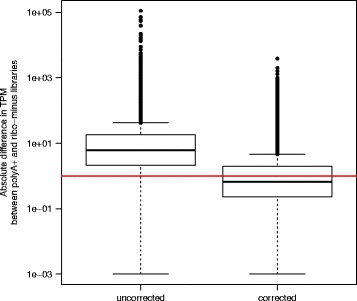
The absolute difference in TPM estimates from polyA+ and ribo-minus libraries was reduced when applying a ratio-based correction to the latter. The line *y* = 1 is shown in red. The data shown is for BMDMs prior to LPS stimulation, quantified using the full Oar v3.1 transcriptome (*n* = 10,667 genes). As data is shown on a logarithmic scale, values of 0 are excluded. To reduce noise, genes with TPM < 1 either before or after correction were excluded

Filtering the reference transcriptome before quantifying expression reduces the difference in TPM estimates caused by differential transcriptome sampling. However, this is at the cost of useful data – filtering the reference transcriptome excludes those transcripts that contribute most to the variance. We considered whether mathematical correction - applied to all transcripts - could resolve TPM underestimation in ribo-minus compared to polyA + libraries (see Methods and Fig. 4). A ratio-based correction of the ribo-minus estimates greatly reduced the absolute difference between polyA+ and ribo-minus TPM in BMDMs (median difference for uncorrected TPMs = 6.06, for corrected TPMs = 0.59, Mann-Whitney U *p* < 2.2 × 10^−16^; Fig. 5). Those genes with greater differences in TPM, even after correction, tended to have fewer unique k-mers (Spearman’s *rho* = −0.34, *p* < 2.2 × 10^−16^), fewer exons (*rho* = −0.17, *p* < 2.2 × 10^−16^), shorter average exon lengths (*rho* = −0.28, *p* < 2.2 × 10^−16^), shorter average transcript lengths (*rho* = −0.35, *p* < 2.2 × 10^−16^), and a greater number of paralogues (*rho* = 0.16, *p* < 2.2 × 10^−16^) (Additional file 1: Table S7). As such, erroneous TPM estimates are more likely when fewer possible reads can be derived from a gene, and when fewer of these reads are unique.

Variance in expression estimates introduced by RNA selection method can also influence downstream analyses. For instance, those protein-coding genes with the greatest absolute difference in uncorrected TPM estimates (Additional file 1: Table S8) are enriched in a variety of biological processes, most notably as structural components of the cytosolic ribosome (Additional file 1: Table S9).

### Transcriptome filtering and TPM correction together minimise differences between polyA+ and ribo-minus libraries

To apply both methods, we devised a two-step pipeline for using Kallisto (see Methods). This involves, as the first step, pseudoaligning all reads to the complete reference transcriptome (that is, assigning reads to transcripts without base-level alignment), parsing this output to create a revised version, and then quantifying expression using that revised reference (the second step). This revised transcriptome was intended to include those transcripts that are absent from the original reference (i.e. accounting for an incomplete annotation), and to exclude misleading or incorrect reference transcripts (i.e. accounting for spurious models in the annotation). Filtering non-expressed isoforms from the reference transcriptome has previously been shown to reduce the false discovery rate in studies of differential transcript usage [44]. When this analysis is applied to a broader sample – such as the range of tissues comprising an expression atlas – unexpressed transcripts would likely be spurious models arising from poor assembly. Alternatively, and more likely in the context of the present data, they could be tissue-specific for a tissue not sampled, or expressed below a detection threshold.

Taken together, these filters increased the proportion of unique k-mers in Kallisto’s index, and by extension the accuracy of TPM estimates.

**Fig. 6:**
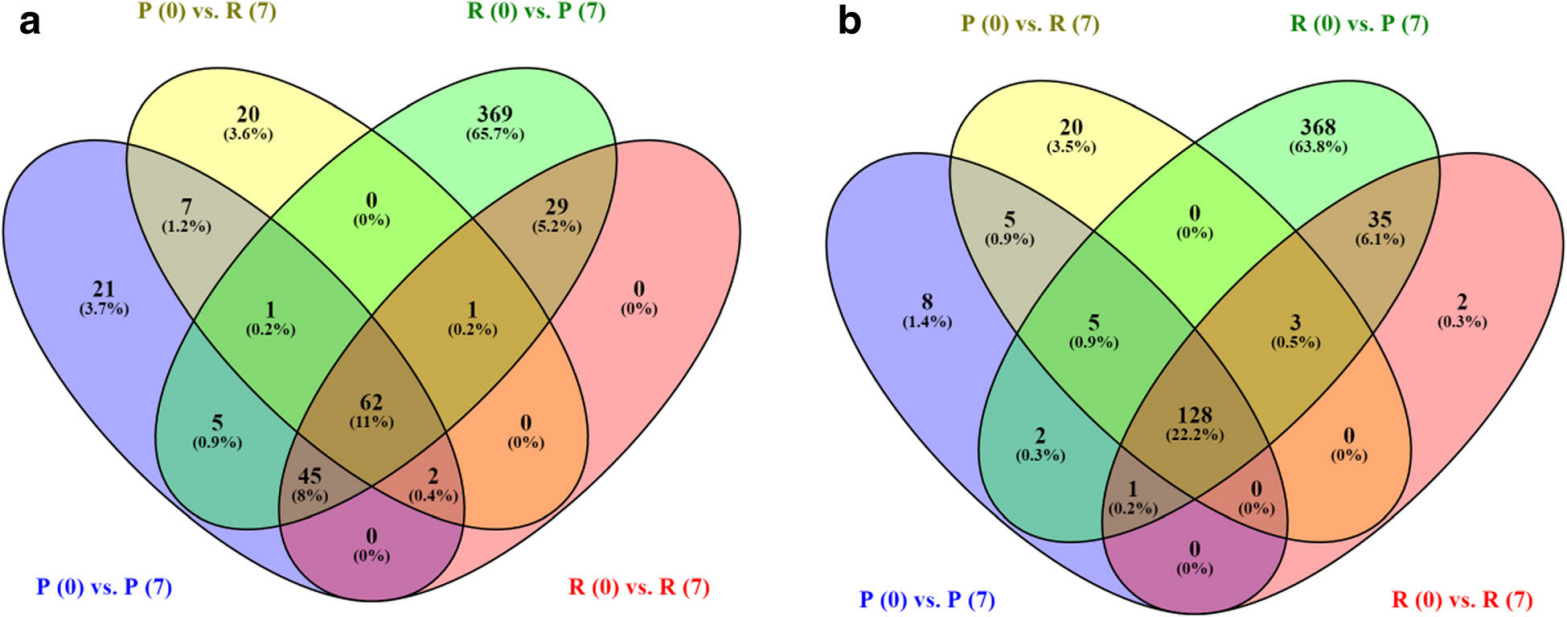
Different library types detect different sets of LPS-inducible transcripts, although a greater number can be captured when merging datasets. Counts are of the number of protein-coding genes with >1 TPM both pre- and post-LPS stimulation (0 and 7 h, respectively) and a log_2_ fold change in expression >2. Data was creating either by (**a**) quantifying expression against the complete Oar v3.1 transcriptome, and (**b**) employing a filtered reference transcriptome for quantifying expression (restricted only to protein-coding genes, excluding those genes not expressed in BMDMs and adding de novo assembled transcripts), and then applying a ratio-based correction to TPM estimates from ribo-minus libraries. After this process of filtering and correction, the number of LPS-inducible genes in the intersection of all sets is doubled. P = polyA+, R = ribo-minus. Figure created using Venny 2.1 [88]

Using this method, we compared the sets of LPS-inducible transcripts detected using each library method, either like with like, or with the reciprocal cross-over (e.g. control polyA+ versus LPS-simulated ribo-minus). The Venn diagram for the four sets is shown in Fig. 6, showing that after filtering the transcriptome and correcting the TPM estimates, the number of LPS-inducible genes in the intersection of all sets is doubled.

This method can also be applied more generally. To demonstrate, we expanded the scope of an existing human tissue expression atlas by merging two publicly available sets of paired-end strand-specific RNA-seq data (113 ribo-minus libraries from the Gingeras lab, CSHL, released as part of the ENCODE Project [NCBI BioProject PRJNA30709] [13], and 13 polyA+ libraries from the Snyder lab, Stanford [GEO accession GSE3605] [45]) (Additional file 1: Table S10). The polyA+ dataset sequences a corresponding set of tissues as those in the larger ribo-minus dataset, and aside from selection method both used broadly similar protocols (the polyA+ libraries are of 100 bp reads sequenced on an Illumina HiSeq2000; the ribo-minus libraries are of 101 bp reads sequenced on an Illumina HiSeq2500).

As a reference transcriptome, we used only the set of multi-exonic protein-coding transcripts (human annotation GRCh38.p7) with a transcript support level of 1 (*n* = 45,239; all splice junctions of the transcript are supported by at least one non-suspect mRNA), 2 (*n* = 42,275; the best supporting mRNA is either flagged as suspect or the support is from multiple ESTs) or 3 (*n* = 35,542; the only support for this transcript is from a single EST). Transcripts with lower support level scores either have no mRNA supporting the model structure or with the best supporting EST being suspect. Single-exon transcripts are also excluded as these do not yet have a transcript support level score. Together, this represents 19,716 genes. Tissue trees were then constructed from the Euclidean distances between gene expression level vectors. These show that prior to TPM correction, the majority of the polyA+ samples group with each other, rather than with the equivalent tissues from the ribo-minus libraries (Additional file 2: Figure S4). This suggests that, in general, library-specific variation confounds a comparative analysis of the two datasets. After correcting TPM estimates as described here, more meaningful biological groupings were obtained for the set of analysed tissues, irrespective of library type (Additional file 2: Figure S5). Furthermore, on a tissue-by-tissue basis, the expression profiles from the two datasets became more tightly correlated after TPM correction (considering those tissues common to both datasets and matched, as closely as possible, by sex and age: the adrenal gland, liver, ovary, sigmoid colon, spleen, and testis; Additional file 2: Figs. S6 to S11, respectively).

## Discussion

Large transcriptomic datasets can be used to infer the function of genes based upon the transcriptional company they keep [46]. The information content of such co-expression networks depends to a large extent upon the size and diversity of biological states sampled; the more states that are sampled, the more stringently one can state that a pair of genes share strict co-expression. With the increasing reproducibility of microarray platforms, many studies have combined data from multiple sources – for example, in a global meta-analysis of human and mouse datasets [47]. Sequence-based expression profiling has emerged rapidly as an alternative to microarrays. The FANTOM5 consortium produced comprehensive expression atlases for mouse and human based upon tag sequencing of 5′ ends (cap analysis gene expression; CAGE) [48, 49]. To date, there have been few efforts to produce meta-datasets from RNA-seq data. A recent study of human lung cancer provides an example of the potential power of combining data [50], although the focus was on the detection of novel fusion transcripts rather than expression quantitation.

In conjunction with decreasing sequencing costs, the number of available RNA-seq datasets has increased substantially in recent years, with gene-level expression estimates available for many species across a range of tissues, cell lines, and developmental stages.

In order to combine microarray datasets from different laboratories, the data must first be normalized and quality-checked [47]. This study outlined a two-part processing pipeline that would allow the combination of RNA-seq data generated from either the same or different cell types and tissues but with different approaches and from different laboratories. Although simple to implement, it has specific requirements and makes several assumptions.

Central to this approach is the transcript quantification tool Kallisto [31], which builds an index of k-mers from a set of reference transcripts. RNA-seq reads are sheared into k-mers, with exact matches of these k-mers to the index used to quantify expression. Use of Kallisto assumes the reference transcriptome is sufficiently high quality for generating a k-mer index: poorer quality annotations will be more likely to have missing transcript models (which Kallisto will not detect) and miscalled bases (such that k-mers will not exactly match the reads, skewing each transcript’s expression level estimate). Incomplete annotation catalogues are known to negatively affect the detection of differential transcript usage [44].

A means of distinguishing high from low-quality transcripts, using transcript support level (TSL) scores, is only available for limited model species (human and mouse), although this is likely to change rapidly as major animal genomes are completed to much higher quality and supported by deep transcriptomic data. TSL scores have been assigned to all multi-exon GENCODE annotations and evaluate the level of support for a transcript across its full length. The highest scoring transcripts are those with all introns supported by an mRNA alignment to each splice junction [51].

In principle, a reference transcriptome could be derived from the reads directly, by performing a de novo assembly. In practice, this is unreasonable, effectively trading one computational bottleneck for another – Kallisto is quick to quantify expression (~30 million reads can be processed in <10 min [40]) but confidence in its estimates depend on an accurate reference transcriptome, the creation of which is slow (for instance, 4 million reads can be processed in ~60-90 min by the Trinity assembler [52]). Once created, a de novo transcript assembly will also need annotating, as not all isoforms can be considered separately: some may instead be variants of previously annotated genes [44]. Within the proposed two-step pipeline, the ambiguity of the de novo transcripts is minimised (along with the processing time required to create them) because only unmappable reads are input to the assembler. These reads are unlikely to be assembled into variants of an existing gene because if such a gene existed, Kallisto would have mapped reads to it.

Based upon our analysis of much larger datasets in the sheep atlas, the variance introduced by the RNA selection method is much greater than from any other batch effect, although these must still be accounted for as a matter of best practice (see review [21]). Batch effects are inevitable unless the entire set of data comprises a single batch [53]. This is implausible for a given large RNA-seq project, such as an expression atlas – data will inevitably be collected over a lengthy period, introducing the confounding effects of differing laboratory conditions, personnel, equipment, reagents and preparation method (see review [54]). Although batch effects are widespread in RNA-seq studies [55], numerous design strategies – such as replication (to minimise technical variation), randomisation and blocking (of samples assigned to sequencing lanes) – are routinely used to minimise their impact [56, 57]. Technical variation is of particular importance with RNA-seq as the sampling fraction of the total number of molecules per sequencing lane is small (< 0.005%) [58]. The likelihood of detecting genes, particularly lowly expressed genes, is influenced by sequencing depth, with variance in gene detection consistent with sampling error. As such, a number of genes will likely remain below detection thresholds because of their low expression level relative to sample size (i.e. sequencing depth), estimated to be up to 10% of the genes per library in many studies [59].

Based upon 5′ end tag sequencing, expressed transcripts in mammalian cells follow a power law distribution [60], which means that the large majority of RNA-seq reads in any dataset derive from the small number of most highly-expressed transcripts. At the lower end of the expression profile, there is a bimodal distribution of transcript detection, where the very low detection of transcripts correlates with the absence of detectable functional chromatin marks [61]. Transcripts with low TPMs will be particularly susceptible to factors that could skew these estimates. As demonstrated by our analysis, lowly expressed transcripts are differentially captured by polyA+ and ribo-minus RNA-seq libraries. Arguably, when one is dealing with relatively pure populations of cells, these transcripts are very unlikely to have a functional impact, being less than 1 transcript per cell, but in tissues they might be more abundant in a subset of rare cells. In the absence of a way to increase read length (which would reduce the likelihood of multiple mapping) or read depth (which would more accurately detect lowly expressed transcripts), we filtered the set of reference transcripts (against which TPM is quantified) to increase the proportion of unique k-mers within it. In conjunction with a mathematical correction for library-specific noise, this also minimised the likelihood that reads will map to multiple loci. This improved the accuracy of TPM estimation to the extent that equivalent estimates can be made from polyA+ and ribo-minus libraries, most notably for protein-coding genes (demonstrated in Additional file 2: Figs. S2 and S3).

## Conclusion

Although polyA-selected and rRNA-depleted libraries capture different fractions of the transcriptome, a combination of reference transcriptome filtering and a ratio-based correction can generate equivalent expression levels from both. This could conceivably assist in the novel re-use of existing RNA-seq data.

## Methods

### Animals

Six adult Scottish Blackface x Texel sheep of approximately 2 years of age (3 male and 3 female) were euthanized by the schedule 1 cull method: electrocution followed by exsanguination. All animal work was conducted in accordance with guidelines of the Roslin Institute and the University of Edinburgh and carried out under the regulations of the Animals (Scientific Procedures) Act 1986. Approval was obtained from The Roslin Institute’s and the University of Edinburgh’s Protocols and Ethics Committees. All data derived from these animals, as detailed below, constitute part of a transcriptional atlas developed at the Roslin Institute [36]. Details of all samples collected for this atlas are included in the BioSamples database (http://www.ebi.ac.uk/biosamples) under submission identifier GSB-718 (group SAMEG317052).

### Cell isolation and stimulation with LPS

Bone marrow cells were isolated from 10 posterior ribs, on the day of euthanasia, as detailed for pig [62]. Bone marrow derived macrophages (BMDMs) were obtained by culturing bone marrow cells for 7 days in the presence of rhCSF-1 (10^4^ U/ml; a gift of Chiron, Emeryville, CA) with 20% sheep serum (Sigma Aldrich) on 100 mm^2^ sterile petri dishes, as described previously for pig [62]. The resulting macrophages were detached by vigorous washing with medium using a syringe and 18-g needle, then washed, counted, and seeded in tissue culture plates overnight at 10^6^ cells/ml in rhCSF-1-containing medium prior to challenge with LPS. The cells were treated with LPS from *Salmonella enterica* serotype minnesota Re 595 (L9764; Sigma-Aldrich) at a final concentration of 100 ng/ml (also as previously described in pig [62]) and then harvested at 0 and 7 h post-LPS treatment.

### RNA extraction, library preparation and sequencing

RNA was extracted according to the method described in [36]. Cells were harvested into 1 ml of TRIzol® reagent (Thermo Fisher Scientific) and equilibrated to room temperature for 5 min. 200 μl BCP (1-bromo-3-chloropropane) (Sigma Aldrich) was added and the sample shaken vigorously for 15 s then incubated for 3 min at room temperature. The sample was then centrifuged for 15 min at 12,000 g, at 4 °C, to separate into a clear upper aqueous layer (which contains RNA), and red lower organic layers (which contain the DNA and proteins). So as to avoid precipitating the RNA, the upper aqueous phase was then removed and cleaned using the RNeasy Mini Kit (Qiagen) with an on-column DNase treatment, in accordance with manufacturer’s guidelines. RNA quantity was estimated using a Qubit RNA BR Assay kit (Invitrogen) and the RNA integrity estimated on an Agilent 2200 Tapestation System to assess quality using the RIN^e^ value. Only samples with RIN^e^ > 8 were sequenced. All RNA-seq libraries were prepared by Edinburgh Genomics using both rRNA-depleted (Illumina total RNA TruSeq) and polyA-selected (Illumina mRNA TruSeq) protocols and sequenced on an Illumina HiSeq 2500.

These samples were sequenced as part of an ovine transcriptional atlas of multiple tissues and primary cells, developed at the Roslin Institute [36] – rRNA-depleted samples were prepared at a depth of >100 million strand-specific 125 bp paired-end reads per sample; polyA-selected samples at a depth of >25 million. The raw data is deposited in the European Nucleotide Archive under study accession PRJEB19199﻿ (http://www.ebi.ac.uk/ena/data/view/PRJEB19199). Prior to analysis, all data was randomly downsampled to exactly 25 million reads using seqtk (https://github.com/lh3/seqtk, downloaded 29th November 2016).

### Expression quantification and defining a reference transcriptome

The initial basis for the Kallisto index was broad: the complete set of protein-coding cDNAs (ftp://ftp.ensembl.org/pub/release-86/fasta/ovis_aries/cdna/Ovis_aries.Oar_v3.1.cdna.all.fa.gz; *n* = 23,113 transcripts [22,823 protein-coding, 247 pseudogene, 43 processed pseudogene]) combined with the set of non-protein coding transcripts (*n* = 6005), obtained from Ensembl BioMart (filtered by type: lincRNA, miRNA, misc_RNA, Mt_rRNA, Mt_tRNA, rRNA, snoRNA, snRNA) [63]. Taken together, this represents 27,054 genes (29,118 transcripts) and 42,812,386 k-mers.

To create an index with a greater proportion of unique k-mers, we identified those reads that Kallisto could not align (for each sample’s pseudobam file, using SAMtools v1.3 with parameter -f 4 [64]). These reads were then assembled de novo using the Trinity assembler, version r20140717 [52, 65] (which also makes use of the k-mer counting algorithm Jellyfish v2.2.5 [66] and the aligner Bowtie v1.1.2 [67]). We filtered these assembled transcripts to retain only those that could be robustly annotated, excluding those whose coding sequence (CDS) is unlikely to encode a protein (as these are less likely to be real). The following criteria were applied: (a) the transcript must encode an open reading frame (ORF) of at least 100 amino acids (using TransDecoder v2.1.0 [52] with LongOrfs parameters -S and -m 100), which (b) must contain a known protein domain (based on a search, by HMMER v3.1b2 [68] with E-value 1e-5, of the Pfam database of protein families, v29.0 [69]), and (c) must share homology with a known peptide (based on a search, by BLAST+ v2.3.0 [70], of the Swiss-Prot [71, 72] March 2016 release: blastp [73] with parameters -max_target_seqs 100 and -evalue 1e-25). Results were filtered to remove those Swiss-Prot entries that are protein fragments, and those whose PE (protein existence) code is not either 1 or 2 (‘experimental evidence at the protein level’ [such as by Edman sequencing, mass spectrometry, X-ray or NMR structure, evidence of protein-protein interaction or antibody-based detection] and ‘experimental evidence at the transcript level’ [such as the existence of cDNA, RT-PCR or Northern blots], respectively).

We then associated, per sample, each CDS with its best hit Swiss-Prot accession: the hit with the longest alignment length, after excluding all hits with <50% identity. This alignment was validated using the ‘needle’ module of the EMBOSS suite [74] with parameters -gapopen 10.0 and -gapextend 0.5 (‘needle’ implements the Needleman-Wunsch algorithm, i.e. provides a global, end-to-end, alignment rather than the local alignment of blastp). Global alignments were made between the peptide encoded by the predicted ORF and its best Swiss-Prot hit. Those that had <50% identity were discarded.

Gene symbols were then assigned according to the best Swiss-Prot hit unless that symbol, or one of its synonyms (according to NCBI: ftp://ftp.ncbi.nlm.nih.gov/gene/DATA/gene_info.gz, downloaded 7th April 2016), was already present in the reference annotation.

Finally, we assessed these sequences for coding potential using the online tool CPAT v1.2.2 [75] (http://lilab.research.bcm.edu/cpat/index.php, accessed 13th December 2016). CPAT assigns a coding probability to sequences based on both Fickett TESTCODE score (which distinguishes protein-coding RNA from ncRNA according to nucleotide composition and codon usage bias) [76] and differential hexamer usage (which, given the dependence between adjacent amino acids in a peptide, discriminates coding from non-coding sequences with high accuracy) [77]. CPAT significance cutoffs were applied based on the human hg19 assembly (as the sheep assembly is not explicitly supported). After excluding those with low coding probability (in the case of a human model, if *p* < 0.364), 7 sequences were retained (Additional file 1: Table S11). These are indicative of novel CDS and were added to the revised transcriptome (contributing 3163 new k-mers; Additional file 1: Table S2).

We note that these unmapped reads were obtained from a single cell type in two states, and that with a broader range of samples (such as in an expression atlas), a greater number of transcripts could conceivably be reconstructed.

In addition, we identified those transcripts that, after the first use of Kallisto, have zero expression across all samples, excluding these from the revised transcriptome (*n* = 8549, representing 8260 genes) (Additional file 1: Table S1).

### Ratio-based correction for ribo-minus TPM

We calculated, per gene, the ratio of median TPM across all polyA+ libraries to median TPM across all ribo-minus libraries. We assumed that this ratio was not skewed by library-specific expression and that deviations from 1 reflected the variance introduced by library type. Given a set of genes with expression estimated using both polyA+ and ribo-minus libraries, all ribo-minus TPMs were multiplied by this ratio.

### GO term enrichment

For the subset of protein-coding genes with the largest absolute (uncorrected) difference in TPM between polyA+ and ribo-minus libraries (the top 5% of the distribution), enrichment for Gene Ontology (GO) terms [78] was assessed using the R package topGO [79]. This utilises the ‘weight’ algorithm to account for the nested structure of the GO tree, with correction for multiple hypothesis testing intrinsic to the approach [80]. topGO requires a reference set of GO terms, built manually by obtaining the Oar_v3.1 set from Ensembl BioMart v88 [81] and filtering to remove those with evidence codes NAS (non-traceable author statement) or ND (no biological data available). We further excluded from analysis those GO terms annotated to fewer than 50 genes in the genome, and any term where the observed number of genes annotated to it in the subset does not exceed the expected number by 2-fold or greater.

### Statistical and phylogenetic analyses

Dendrograms, made using the Euclidean distances between expression vectors, were constructed with MEGA v7.0.14 [82] using the neighbour-joining method. All statistical analyses were performed in R v3.3.1 [83]. Cliff’s delta [84, 85], a non-parametric effect size estimator, is used to interpret the results of T-tests. This measure relies on the concept of dominance (which refers to the degree of overlap between distributions) rather than means (as in conventional effect size indices such as Cohen’s *d*) and so is more robust when distributions are skewed. Cliff’s delta, at a confidence level of 95%, was estimated using the R package ‘effsize’ [86]. These estimates are bound in the interval (−1,1), with extreme values indicating minimal or no overlap between the two groups (interpretable as group 1 < < group2 and group 1 > > group 2, respectively). Differences between groups with an associated |delta| of <0.147 can be considered negligible, as in [87]. Conversely, |delta| > = 0.60 can be considered a large effect.

## Additional files

Additional file 1: Table S1. Gene expression level for ovine BMDMs pre- and post-LPS stimulation, quantified against the full Oar v3.1 reference transcriptome using polyA-selected and rRNA-depleted libraries. **Table S2.** Comparison of polyA+ and ribo-minus TPM distributions with different reference transcriptomes. **Table S3.** Genes detected in both polyA-selected and rRNA-depleted libraries, if quantified against the full Oar v3.1 reference transcriptome. **Table S4.** Genes detected only in polyA-selected libraries, if quantified against the full Oar v3.1 reference transcriptome. **Table S5**. Genes detected only in rRNA-depleted libraries, if quantified against the full Oar v3.1 reference transcriptome. **Table S6.** Number of pseudoalignments per sample, if quantified against the full Oar v3.1 reference transcriptome. **Table S7.** Differences in expression between polyA-selected and rRNA-depleted libraries when varying the reference transcriptome. **Table S8.** Protein-coding genes with the largest absolute difference between polyA+ and ribo-minus TPM. **Table S9.** GO term enrichment for the set of protein-coding genes with the largest absolute difference between polyA+ and ribo-minus TPM. **Table S10.** Human gene expression meta-atlas, generated using polyA+ and rRNA-depleted ENCODE RNA-seq data. **Table S11.** Coding potential of putative novel CDS. (XLSX 32632 kb)

Additional file 2: Figure S1. Variance in expression estimates arising from differential transcriptome sampling by polyA+ and ribo-minus RNA selection methods. **Figure S2.** Variance in TPM estimates by differential transcriptome sampling was effectively negated by the combined use of a filtered reference transcriptome for quantifying expression, and applying a ratio-based correction to the TPM estimates of ribo-minus libraries. **Figure S3.** Reduction in the absolute difference in TPM estimates from polyA+ and ribo-minus libraries when applying a ratio-based correction to the latter. **Figure S4.** Tissue tree constructed from the Euclidean distances between uncorrected TPM vectors for two human RNA-seq datasets sequenced with either polyA+ or ribo-minus libraries. **Figure S5.** Tissue tree constructed from the Euclidean distances between corrected TPM vectors for two human RNA-seq datasets sequenced with either polyA+ or ribo-minus libraries. **Figure S6.** Comparison of TPM estimates in the adrenal gland, as generated for 19,716 human genes using both polyA-selected and rRNA-depleted libraries. **Figure S7.** Comparison of TPM estimates in the liver, as generated for 19,716 human genes using both polyA-selected and rRNA-depleted libraries. **Figure S8.** Comparison of TPM estimates in the ovary, as generated for 19,716 human genes using both polyA-selected and rRNA-depleted libraries. **Figure S9.** Comparison of TPM estimates in the sigmoid colon, as generated for 19,716 human genes using both polyA-selected and rRNA-depleted libraries. **Figure S10.** Comparison of TPM estimates in the spleen, as generated for 19,716 human genes using both polyA-selected and rRNA-depleted libraries. **Figure S11.** Comparison of TPM estimates in the testis, as generated for 19,716 human genes using both polyA-selected and rRNA-depleted libraries. (DOCX 709 kb)

## Abbreviations

BFxT: Scottish Blackface x Texel
BMDM: Bone marrow derived macrophage
CAGE: Cap analysis gene expression
CDS: Coding sequence
lncRNA: Long non-coding RNA
LPS: Lipopolysaccharide
ORF: Open reading frame
polyA-: Non-polyadenylated
polyA+: Polyadenylated
RNA-seq: RNA sequencing
TPM: Transcripts per million
