# Supplementary material for: Integration of quantitated expression estimates from polyA-selected and rRNA-depleted RNA-seq libraries
Source: BMC Bioinformatics. 2017 Jun 13;18:301. doi: 10.1186/s12859-017-1714-9 (PMC5470212; doi:10.1186/s12859-017-1714-9)
Supplement: Supplementary file 2 — Variance in expression estimates arising from differential transcriptome sampling by polyA+ and ribo-minus RNA selection methods. Figure S2. Variance in TPM estimates by differential transcriptome sampling was effectively negated by the combined use of a filtered reference transcriptome for quantifying expression, and applying a ratio-based correction to the TPM estimates of ribo-minus libraries. Figure S3. Reduction in the absolute difference in TPM estimates from polyA+ and ribo-minus libraries when applying a ratio-based correction to the latter. Figure S4. Tissue tree constructed from the Euclidean distances between uncorrected TPM vectors for two human RNA-seq datasets sequenced with either polyA+ or ribo-minus libraries. Figure S5. Tissue tree constructed from the Euclidean distances between corrected TPM vectors for two human RNA-seq datasets sequenced with either polyA+ or ribo-minus libraries. Figure S6. Comparison of TPM estimates in the adrenal gland, as generated for 19,716 human genes using both polyA-selected and rRNA-depleted libraries. Figure S7. Comparison of TPM estimates in the liver, as generated for 19,716 human genes using both polyA-selected and rRNA-depleted libraries. Figure S8. Comparison of TPM estimates in the ovary, as generated for 19,716 human genes using both polyA-selected and rRNA-depleted libraries. Figure S9. Comparison of TPM estimates in the sigmoid colon, as generated for 19,716 human genes using both polyA-selected and rRNA-depleted libraries. Figure S10. Comparison of TPM estimates in the spleen, as generated for 19,716 human genes using both polyA-selected and rRNA-depleted libraries. Figure S11. Comparison of TPM estimates in the testis, as generated for 19,716 human genes using both polyA-selected and rRNA-depleted libraries. (DOCX 709 kb) [file 12859_2017_1714_MOESM2_ESM.docx]

**Integration of quantitated expression estimates from** **polyA-selected and rRNA-depleted RNA-seq libraries**

Stephen J. Bush^1*^, Mary E. B. McCulloch^1^, Kim M. Summers^1^, David A. Hume^1*†^, Emily L. Clark^1†^

^1^ The Roslin Institute and Royal (Dick) School of Veterinary Studies, University of Edinburgh, Easter Bush, Midlothian, EH25 9RG, United Kingdom

* corresponding author

^†^ contributed equally to this work

**SUPPLEMENTARY FIGURES**


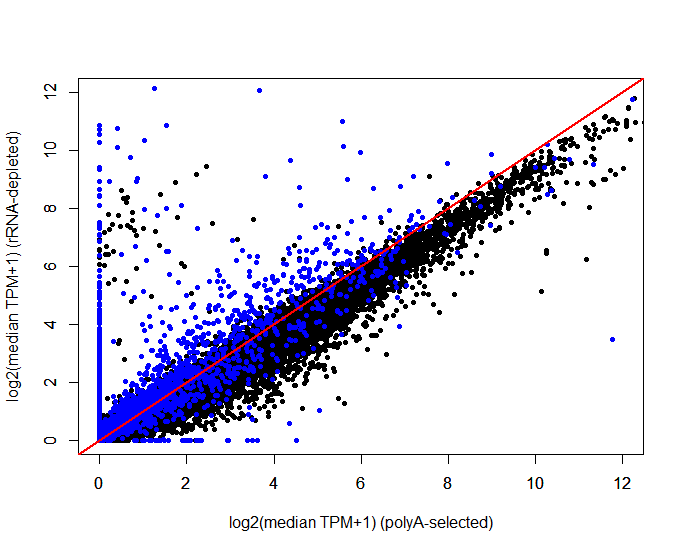


**Supplementary Figure 1.** Variance in expression estimates arising from differential transcriptome sampling by polyA+ and ribo-minus RNA selection methods. Each point is a gene, coloured by type: black points represent protein-coding genes, pseudogenes and processed pseudogenes; blue points represent RNA genes. The line *x* = *y* is shown in red. As ribo-minus libraries capture RNA genes that polyA+ libraries do not (the line vertically bisecting *x* = 0), expression can be systematically underestimated for the remaining, mostly protein-coding, genes. The data shown is for BMDMs 7 hours after LPS stimulation.


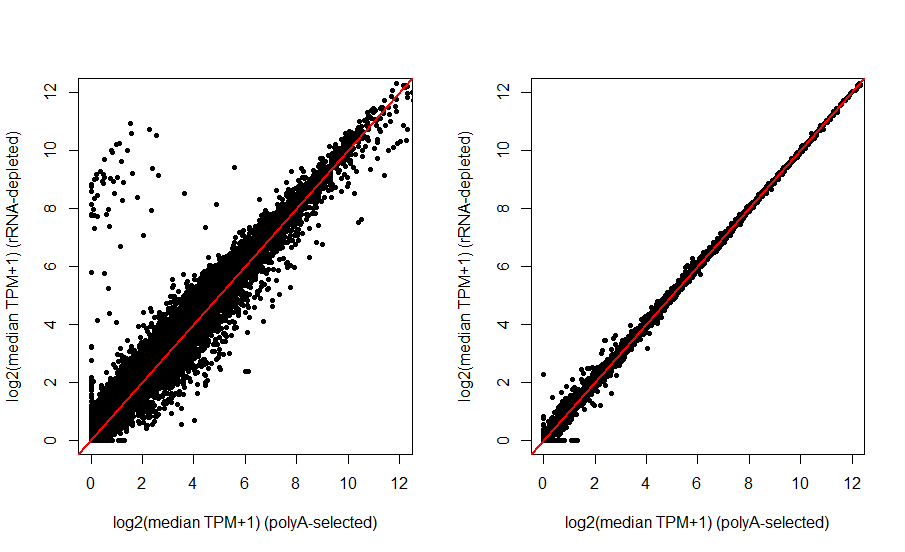


**Supplementary Figure 2.** Variance in TPM estimates by differential transcriptome sampling was effectively negated by the combined use of (a) a filtered reference transcriptome for quantifying expression, and (b) applying a ratio-based correction to the TPM estimates of ribo-minus libraries. The data shown is for BMDMs prior to LPS stimulation, quantified in both cases using the set of protein-coding transcripts (n=20,921 genes). In the rightmost figure, all ribo-minus TPM estimates were multiplied by the ratio of the median TPM across all polyA+ libraries to the median TPM across all ribo-minus libraries. Should the median TPM across all ribo-minus libraries be 0, this ratio was considered 0 also. This correction strengthened the correlation of polyA+ and ribo-minus TPM (Pearson’s *r* = 0.9995, p < 2.2x10^-16^).


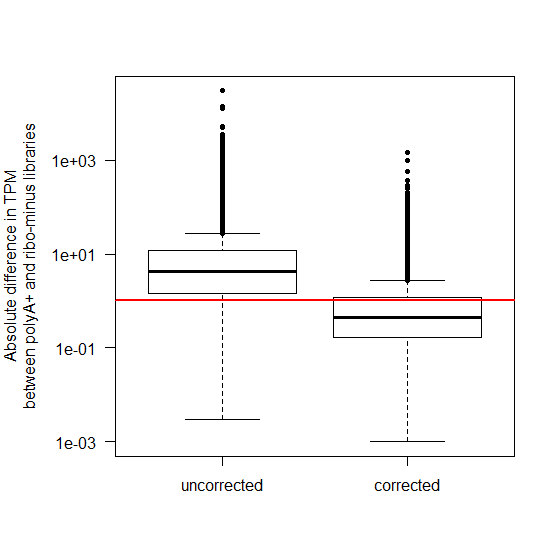


**Supplementary Figure 3.** The absolute difference in TPM estimates from polyA+ and ribo-minus libraries was reduced when applying a ratio-based correction to the latter. The line *y*=1 is shown in red. The data shown is for BMDMs prior to LPS stimulation (n=10,426 genes), quantified using a filtered Oar v3.1 transcriptome. This was created using a two-part pipeline centred on the transcript quantification tool Kallisto. The transcriptome (a) was restricted to protein-coding genes, (b) excluded those genes not expressed in BMDMs, and (c) contained *de novo* assembled transcripts. As data is shown on a logarithmic scale, values of 0 are excluded. To reduce noise, genes with TPM < 1 either before or after correction were excluded. The median difference in TPMs, after correction, is 0.452.

**
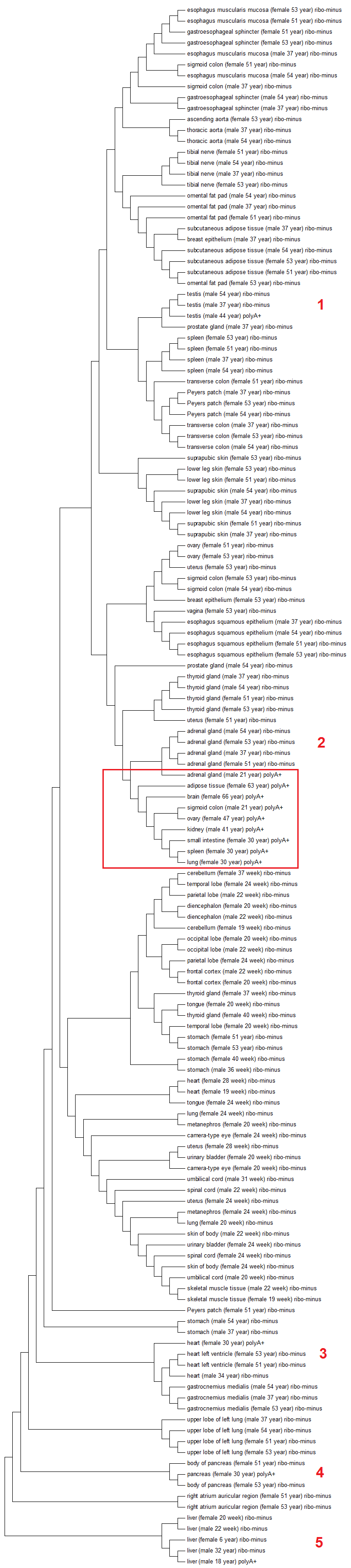
**

**Supplementary Figure 4.** Tissue tree constructed from the Euclidean distances between uncorrected TPM vectors for two human RNA-seq datasets sequenced with either polyA+ or ribo-minus libraries (GEO accession GSE3605 and NCBI BioProject PRJNA30709, respectively). Expression vectors are taken to be the median TPM per gene for all replicates of that tissue, before applying a mathematical correction for library type. The tree was constructed using the neighbour-joining method. Only the topology is shown. The majority of the polyA+ samples group together (red box), rather than with the equivalent tissues from the ribo-minus libraries. Five exceptions are numbered: testis (1), adrenal gland (2), heart (4), pancreas (4) and liver (5). This suggests that, in general, library-specific variation confounds a comparative analysis of the two datasets. A tissue tree created using corrected TPM estimates is available as Supplementary Figure 5.

**
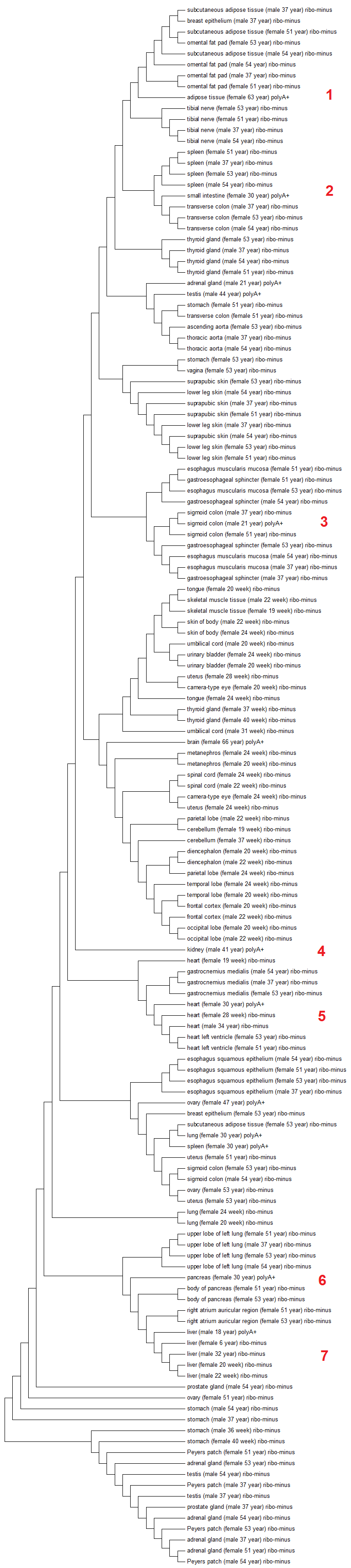
**

**Supplementary Figure 5.** Tissue tree constructed from the Euclidean distances between corrected TPM vectors for two human RNA-seq datasets sequenced with either polyA+ or ribo-minus libraries (GEO accession GSE3605 and NCBI BioProject PRJNA30709, respectively). Expression vectors are taken to be the median TPM per gene for all replicates of that tissue, after applying a mathematical correction for library type. The tree was constructed using the neighbour-joining method. Only the topology is shown. Compared to the tree created from uncorrected TPM estimates (see Supplementary Figure 4), tissues from polyA+ libraries group more meaningfully with those from equivalent ribo-minus libraries, rather than forming a polyA-specific ‘clade’. Numbered examples represent biologically similar groups: (1) polyA+ adipose tissue groups with ribo-minus adipose tissue, omental fat pad and breast epithelium, (2) polyA+ small intestine groups with ribo-minus transverse colon, (3) polyA+ sigmoid colon groups with ribo-minus sigmoid colon, (4) the sole polyA+ kidney sample now constitutes a distinct outgroup (there are no ribo-minus kidney samples with which it could conceivably form a ‘clade’), (5) polyA+ heart groups with ribo-minus heart and gastrocnemius medialis (muscle), (6) polyA+ pancreas groups with ribo-minus pancreas, (7) polyA+ liver groups with ribo-minus liver.


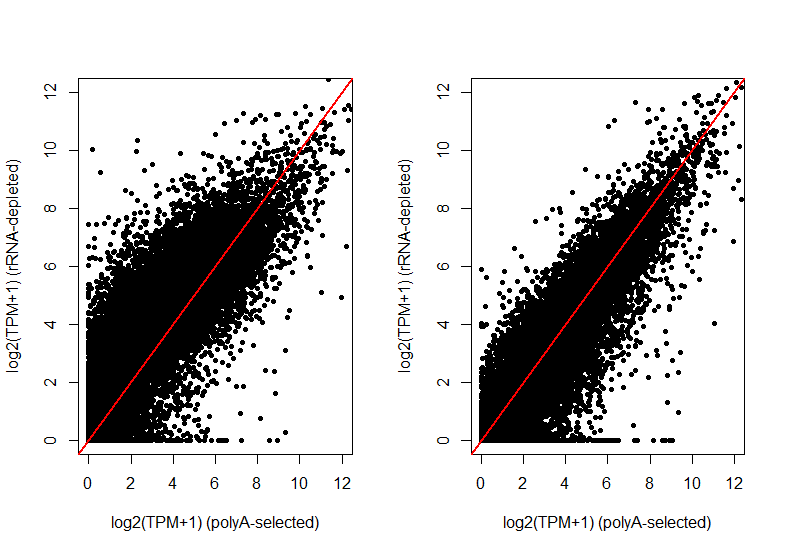
**Supplementary Figure 6.** Comparison of TPM estimates in the adrenal gland, as generated for 19,716 human genes using both polyA-selected and rRNA-depleted libraries (polyA-selected sample: male 21 year, ENCODE experiment ID: ENCSR680AAZ; rRNA-depleted sample: male 37 year, ENCODE experiment ID: ENCBS227VDO). The leftmost graph shows TPM estimates before correction for library type; the rightmost graph shows TPM estimates after correction for library type. After TPM correction, expression estimates are more evenly distributed about the line *y* = *x* (shown in red), with the correlation increasing from Spearman’s *rho* = 0.852 to *rho* = 0.913.


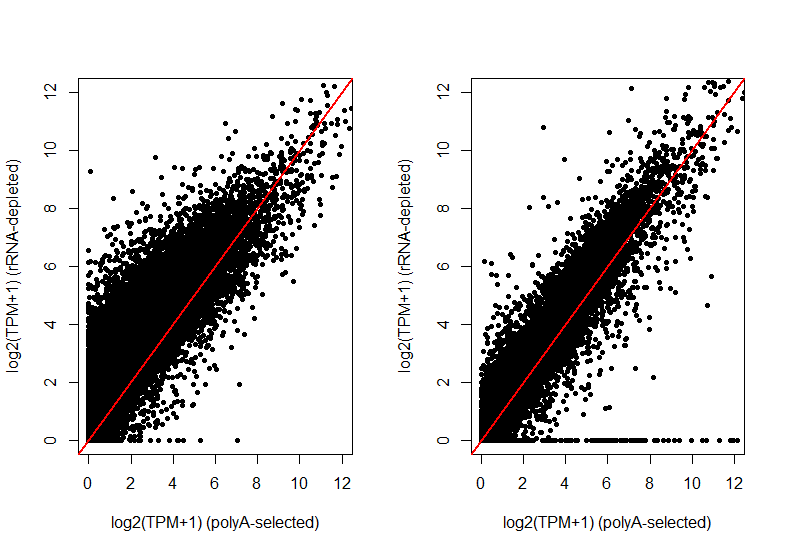


**Supplementary Figure 7.** Comparison of TPM estimates in the liver, as generated for 19,716 human genes using both polyA-selected and rRNA-depleted libraries (polyA-selected sample: male 18 year, ENCODE experiment ID: ENCSR085HNI, rRNA-depleted sample: male 32 year, ENCODE experiment ID: ENCBS046RNA). The leftmost graph shows TPM estimates before correction for library type; the rightmost graph shows TPM estimates after correction for library type. After TPM correction, expression estimates are more evenly distributed about the line *y* = *x* (shown in red), with the correlation increasing from Spearman’s *rho* = 0.897 to *rho* = 0.937.


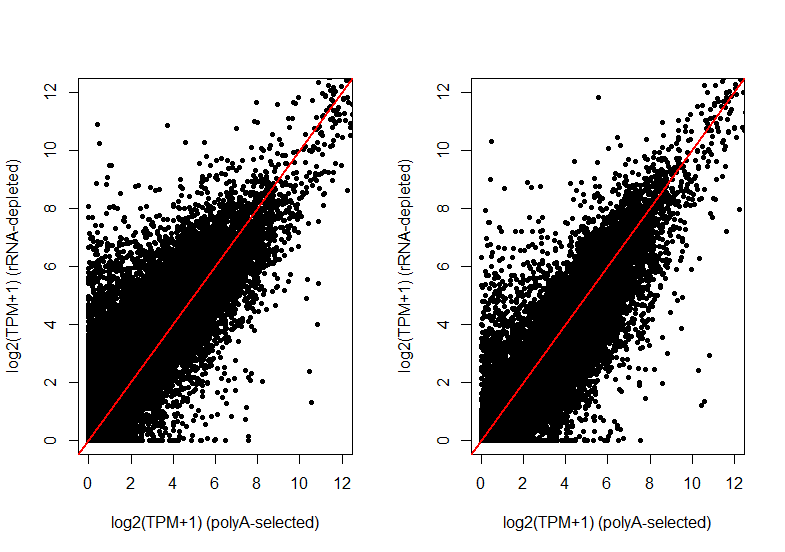


**Supplementary Figure 8.** Comparison of TPM estimates in the ovary, as generated for 19,716 human genes using both polyA-selected and rRNA-depleted libraries (polyA-selected sample: female 47 year, ENCODE experiment ID: ENCSR046XHI, rRNA-depleted sample: female 51 year, ENCODE experiment ID: ENCBS711CPB). The leftmost graph shows TPM estimates before correction for library type; the rightmost graph shows TPM estimates after correction for library type. After TPM correction, expression estimates are more evenly distributed about the line *y* = *x* (shown in red), with the correlation increasing from Spearman’s *rho* = 0.830 to *rho* = 0.891.


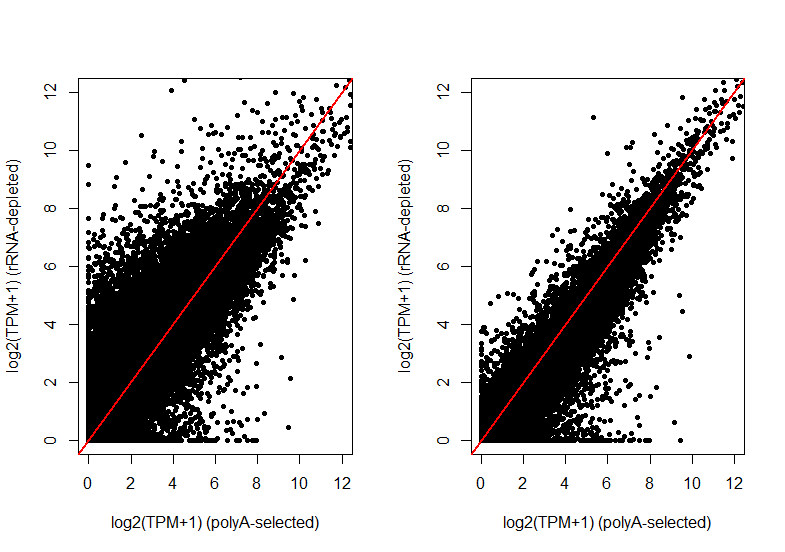


**Supplementary Figure 9.** Comparison of TPM estimates in the sigmoid colon, as generated for 19,716 human genes using both polyA-selected and rRNA-depleted libraries (polyA-selected sample: male 21 year, ENCODE experiment ID: ENCSR270OKS, rRNA-depleted sample: male 37 year, ENCODE experiment ID: ENCBS516KIQ). The leftmost graph shows TPM estimates before correction for library type; the rightmost graph shows TPM estimates after correction for library type. After TPM correction, expression estimates are more evenly distributed about the line *y* = *x* (shown in red), with the correlation increasing from Spearman’s *rho* = 0.810 to *rho* = 0.930.


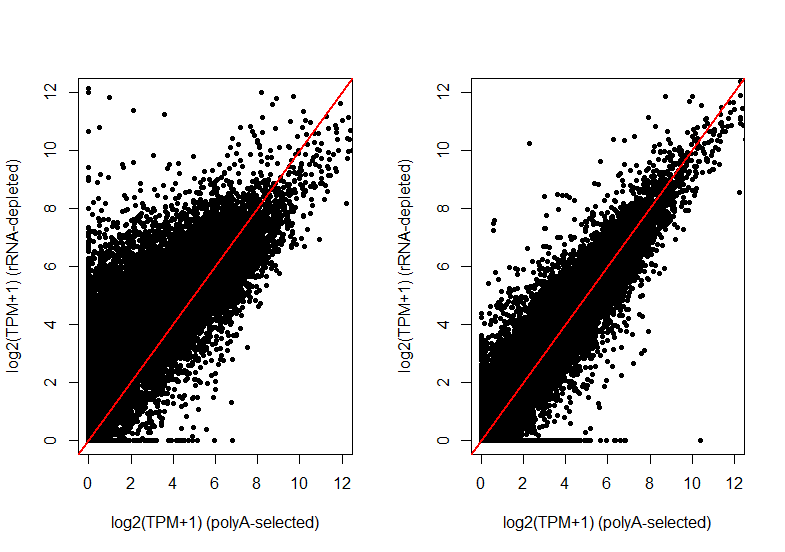


**Supplementary Figure 10.** Comparison of TPM estimates in the spleen, as generated for 19,716 human genes using both polyA-selected and rRNA-depleted libraries (polyA-selected sample: female 30 year, ENCODE experiment ID: ENCSR448VSW, rRNA-depleted sample: female 51 year, ENCODE experiment ID: ENCBS253SDK). The leftmost graph shows TPM estimates before correction for library type; the rightmost graph shows TPM estimates after correction for library type. After TPM correction, expression estimates are more evenly distributed about the line *y* = *x* (shown in red), with the correlation increasing from Spearman’s *rho* = 0.811 to *rho* = 0.935.


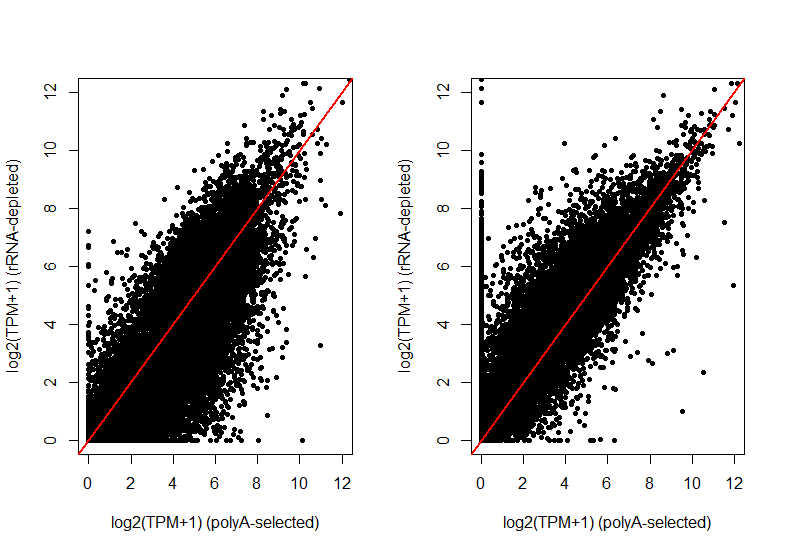


**Supplementary Figure 11.** Comparison of TPM estimates in the testis, as generated for 19,716 human genes using both polyA-selected and rRNA-depleted libraries (polyA-selected sample: male 37 year, ENCODE experiment ID: ENCBS315DHM, rRNA-depleted sample: male 44 year, ENCODE experiment ID: ENCSR693GGB). The leftmost graph shows TPM estimates before correction for library type; the rightmost graph shows TPM estimates after correction for library type. After TPM correction, expression estimates are more evenly distributed about the line *y* = *x* (shown in red), with the correlation increasing from Spearman’s *rho* = 0.796 to *rho* = 0.856.
